# Silent Threats After Surgery: Incidence and Predictors of Deep Vein Thrombosis and Pulmonary Embolism in Orthopedic Patients

**DOI:** 10.3390/diagnostics15182352

**Published:** 2025-09-16

**Authors:** Serkan Aydin, Burhan Kurtulus

**Affiliations:** 1 Department of Orthopedics and Traumatology, Ankara Etlik City Hospital, Ankara 06110, Turkey; 2 Department of Orthopedics and Traumatology, Ankara Diskapi Yildirim Beyazit Education and Research Hospital, Ankara 06110, Turkey; kurtulusburhan@gmail.com

**Keywords:** deep vein thrombosis, pulmonary embolism, orthopedic surgery, risk factors, thromboprophylaxis, biomarkers

## Abstract

**Objectives**: This study aimed to determine the incidence of postoperative deep vein thrombosis (DVT) and pulmonary embolism (PE) following orthopedic surgeries and to identify independent clinical, laboratory, and procedural factors associated with thromboembolic risk. **Materials and Methods**: A retrospective cohort analysis was conducted on 300 patients who underwent elective or emergency orthopedic surgeries (hip/knee arthroplasty, fracture fixation, and spinal procedures) between January 2020 and December 2024 at two tertiary centers. Demographic, clinical, and biochemical data were collected. Patients were stratified into two groups: those who developed DVT/PE and those who did not. Univariate analyses were performed to identify significant factors, and a multivariate logistic regression model with stepwise variable selection was applied in accordance with the events-per-variable (EPV) criterion. Receiver operating characteristic (ROC) curve analyses were conducted to evaluate the discriminative performance of significant predictors. **Results**: Among 300 patients who underwent orthopedic surgery, postoperative deep vein thrombosis (DVT) and/or pulmonary embolism (PE) occurred in 50 cases (16.7%). Patients who developed thromboembolic events were older (72.5 ± 8.7 vs. 65.2 ± 10.1 years, *p* < 0.001), had higher body mass index (32.1 ± 5.3 vs. 28.3 ± 4.5 kg/m^2^, *p* < 0.001), and showed a greater prevalence of diabetes mellitus (40% vs. 20%, *p* < 0.01) and chronic kidney disease (24% vs. 10%, *p* < 0.001) compared to those without DVT/PE. Laboratory analyses revealed significantly elevated neutrophil count, D-dimer, C-reactive protein (CRP), glucose, and troponin levels in the DVT/PE group. In the stepwise multivariate logistic regression model, age (OR = 1.44, *p* = 0.003), diabetes mellitus (OR = 2.88, *p* = 0.046), chronic kidney disease (OR = 2.33, *p* = 0.014), D-dimer (OR = 2.15, *p* = 0.019), and immobilization duration (OR = 2.21, *p* = 0.028) emerged as independent predictors of thromboembolic events. ROC analysis revealed that D-dimer > 0.9 mg/L had the highest discriminative performance (AUC = 0.89, sensitivity 88%, specificity 84%, *p* = 0.003), followed by troponin > 0.5 U/L (AUC = 0.86, *p* = 0.005), immobilization > 3 days (AUC = 0.82, *p* = 0.012), and age > 65 years (AUC = 0.74, *p* = 0.021). **Conclusions**: DVT and PE remain significant postoperative complications with a multifactorial etiology in orthopedic surgeries. Advanced age, comorbidities (such as diabetes mellitus and chronic kidney disease), and elevated inflammatory and metabolic markers (including neutrophil count, glucose, CRP, and D-dimer), together with procedural factors like prolonged immobilization, were identified as independent risk factors. Early recognition of these high-risk features and implementation of individualized prophylaxis strategies may improve postoperative outcomes and reduce thromboembolic risk.

## 1. Introduction

Orthopedic surgeries, particularly lower extremity procedures, carry a high risk of thromboembolic complications in the postoperative period [1,2,3,4]. Deep vein thrombosis (DVT) and pulmonary embolism (PE) are the most feared complications, as they contribute significantly to postoperative morbidity and mortality [5,6]. Procedures such as total hip arthroplasty, total knee arthroplasty, fracture surgeries requiring prolonged immobilization, and spinal operations are particularly associated with increased risk [7,8,9,10].

The mechanisms underlying venous thromboembolism (VTE) are well described by Virchow’s triad: venous stasis, endothelial injury, and hypercoagulability [11]. Orthopedic surgery promotes each of these processes. Prolonged immobilization following surgery exacerbates venous stasis, while direct trauma during surgical manipulation contributes to endothelial injury [12]. In addition, patient-related factors such as obesity, smoking, diabetes mellitus, chronic kidney disease, and inherited thrombophilias amplify hypercoagulability and predispose patients to thrombotic events [13].

Clinically, DVT and PE often present with nonspecific or subtle findings, which complicates timely diagnosis. DVT may manifest as leg swelling, pain, or tenderness, while PE can present with dyspnea, chest pain, or even sudden hemodynamic collapse [14,15]. Because of these diagnostic challenges, preventive measures are emphasized in perioperative care. Mechanical methods such as compression stockings and intermittent pneumatic devices, along with pharmacologic thromboprophylaxis using low-molecular-weight heparin (LMWH) or direct oral anticoagulants (DOACs), are widely recommended [14,15,16,17]. Despite these preventive strategies, thromboembolic events still occur, underscoring the need for improved risk prediction [18].

Previous research has primarily investigated the general incidence and risk factors for VTE in orthopedic surgery, or the efficacy of various prophylactic regimens [1,2,3,4,17]. However, limited studies have evaluated the combined role of demographic, metabolic, inflammatory, and procedural factors in shaping thromboembolic risk. In particular, biomarkers such as D-dimer, C-reactive protein (CRP), and leukocyte subsets, along with perioperative variables including immobilization duration and surgery length, may provide additional predictive value but remain underexplored in integrated models [5,13]. By incorporating inflammatory and metabolic biomarkers alongside procedural risk factors, this study sought to contribute to more precise risk stratification and to support the development of individualized thromboprophylaxis strategies [19,20].

Therefore, the present study aimed to determine the incidence of postoperative DVT and PE in patients undergoing orthopedic surgery and to identify independent clinical, laboratory, and procedural predictors of these complications.

## 2. Materials and Methods

### 2.1. Study Design and Patient Selection

This study was conducted as a retrospective observational cohort analysis of patients who underwent orthopedic surgery at two tertiary care centers between January 2020 and December 2024. Eligible procedures included hip, knee, and ankle arthroplasties, fracture fixations, and spinal surgeries, while patients undergoing arthroscopies, minor interventions, or operations with open vascular injuries were excluded. Based on postoperative outcomes documented in hospital records, patients were stratified into two groups: those who developed thromboembolic complications (DVT/PE [+]) and those who did not (DVT/PE [−]). The diagnosis of DVT was confirmed by lower extremity venous Doppler ultrasonography, whereas PE was verified using thoracic CT angiography. All group allocations were determined retrospectively from clinical and imaging data.

### 2.2. Data Collection

Demographic data (age, sex, body mass index—BMI), comorbidities (such as diabetes mellitus, hypertension, chronic kidney disease, cancer, and hematological disorders like thrombophilia), surgical duration, type of operation, anesthesia method, duration of immobilization, length of hospital stay, and prophylactic interventions (mechanical and pharmacological thromboprophylaxis) were retrospectively obtained from patient files and the hospital information system. Procalcitonin was not part of routine preoperative testing; it was measured postoperatively only in patients with clinical suspicion of infection or systemic inflammation.

### 2.3. Diagnosis of DVT and PE

In the postoperative period, the diagnosis of DVT was confirmed via lower extremity venous Doppler ultrasonography. PE was diagnosed based on clinical suspicion and confirmed using thoracic computed tomography angiography (CT angiography). All diagnoses of DVT and PE were finalized by the relevant specialist physicians.

### 2.4. Assessment of Risk Factors

**Figure 1:**
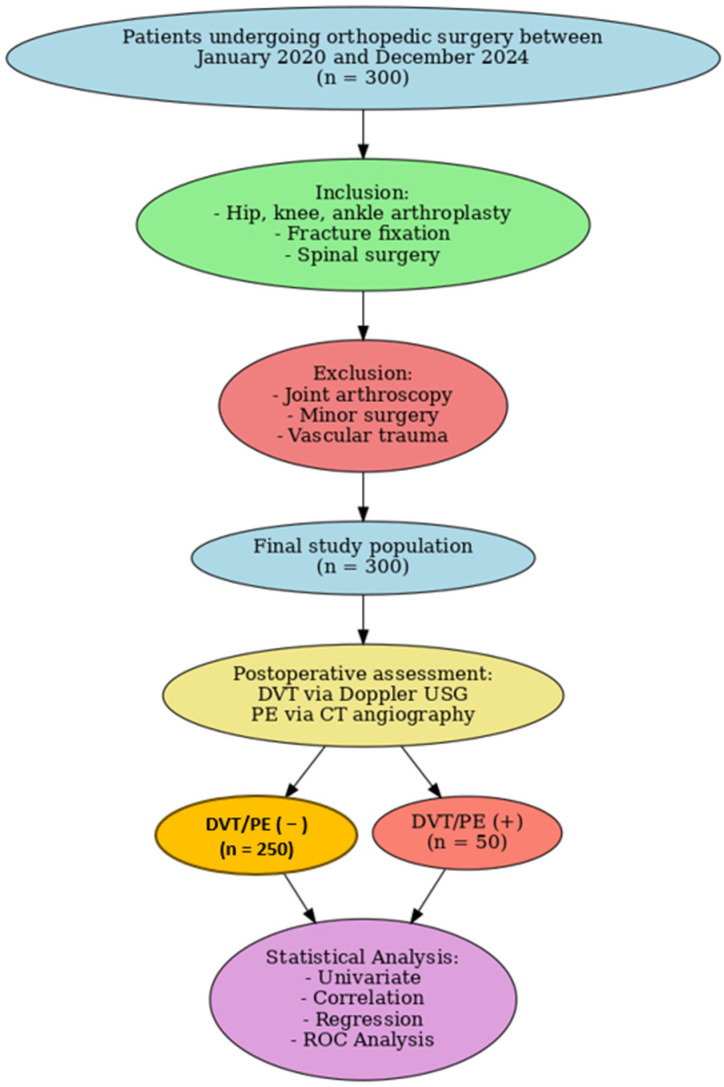
Flowchart of retrospective patient selection and grouping. A total of 300 orthopedic surgery patients (January 2020–December 2024) were classified as DVT/PE (+) if confirmed by imaging, or DVT/PE (−) if no thromboembolic event was documented.

Patients who developed DVT or PE were compared with those who did not in terms of demographic, clinical, and surgical variables. Independent variables including age, sex, BMI, duration of surgery, immobilization time, presence of comorbidities, prophylactic interventions, and laboratory parameters were analyzed to identify factors associated with thromboembolic events (Figure 1).

### 2.5. Statistical Analysis

Data analysis was performed using SPSS version 27.0 (IBM Corp., Armonk, NY, USA). Continuous variables were presented as mean ± standard deviation (SD) or median (interquartile range, IQR) depending on the normality of distribution, while categorical variables were expressed as frequencies and percentages. The normality of data distribution was assessed using the Kolmogorov–Smirnov test. Differences between the DVT/PE (+) and DVT/PE (−) groups were evaluated using the independent samples *t*-test or the Mann–Whitney U test for continuous variables, and the chi-square test for categorical variables. A *p*-value < 0.05 was considered statistically significant. Pearson correlation analysis was used to assess the relationship between surgery duration and immobilization duration with demographic, clinical, and laboratory parameters in patients who developed DVT/PE. Multivariate logistic regression analysis was performed using a stepwise selection approach, limiting the number of predictors according to the events-per-variable (EPV) criterion. Five independent predictors were retained in the final model (age, diabetes mellitus, chronic kidney disease, D-dimer, and immobilization duration). Odds ratios (ORs) and 95% confidence intervals (CIs) were calculated. Receiver Operating Characteristic (ROC) curve analysis was used to evaluate the diagnostic performance of significant parameters in predicting DVT/PE. The area under the curve (AUC), optimal cut-off values, sensitivity, and specificity were calculated for each parameter. A *p*-value < 0.05 was considered statistically significant.

## 3. Results

A total of 300 patients were included in the study, with 86 patients from Lokman Hekim Private Hospital and 214 patients from Etlik City Hospital. A comparison of the sociodemographic characteristics and laboratory findings of patients is shown in Table 1. Fifty patients (16.7%) developed postoperative DVT or PE, and 250 patients (83.3%) did not experience any thromboembolic complications. Patients in the DVT/PE (+) group were significantly older compared to the DVT/PE (−) group (72.5 ± 8.7 vs. 65.2 ± 10.1 years, *p* < 0.001). Male gender was more common among patients who developed DVT/PE (66%) than those who did not (55.2%), although the difference was not statistically significant. BMI was significantly higher in the DVT/PE (+) group (32.1 ± 5.3 vs. 28.3 ± 4.5 kg/m^2^, *p* < 0.001). Comorbidities such as diabetes mellitus (40% vs. 20%, *p* < 0.01), hypertension (50% vs. 35.2%, *p* < 0.01), and chronic kidney disease (24% vs. 10%, *p* < 0.001) were more frequent in patients with DVT/PE. The rate of prophylactic anticoagulation was significantly lower in patients who developed DVT/PE (46% vs. 85.2%, *p* < 0.001).

Laboratory parameters also showed significant differences. Patients with DVT/PE had higher white blood cell count (9.2 ± 2.0 vs. 7.5 ± 1.8 × 10^3^/μL, *p* < 0.01), RDW (15.4 ± 1.5% vs. 13.1 ± 1.2%, *p* < 0.001), platelet count (280 ± 75 vs. 250 ± 60 × 10^3^/μL, *p* < 0.05), neutrophil count (6.1 ± 1.5 vs. 4.5 ± 1.2 × 10^3^/μL, *p* < 0.01), MPV (11.2 ± 1.8 vs. 9.8 ± 1.5 fL, *p* < 0.05), and monocyte count (0.9 ± 0.3 vs. 0.6 ± 0.2 × 10^3^/μL, *p* < 0.001). Conversely, lymphocyte count was lower in the DVT/PE (+) group (1.2 ± 0.4 vs. 1.8 ± 0.6 × 10^3^/μL, *p* < 0.01).

Biochemical markers such as glucose, creatinine, BUN, and CRP were significantly higher in patients with thromboembolic events. Notably, D-dimer (1.8 ± 0.6 vs. 0.5 ± 0.3 mg/L, *p* < 0.001), CRP (6.5 ± 2.3 vs. 3.2 ± 1.1 mg/L, *p* < 0.001), and procalcitonin levels (0.10 ± 0.04 vs. 0.04 ± 0.02 ng/L, *p* < 0.01) were markedly elevated in the DVT/PE group.

DVT was observed in all 50 patients in the DVT/PE (+) group (100%), while PE occurred in 25 of them (50%). Surgery type distribution showed a significant difference (*p* = 0.04), with total hip/knee arthroplasty patients more frequently experiencing thromboembolic events compared to others. Mean surgery duration was longer in the DVT/PE (+) group (135 ± 30 min vs. 110 ± 25 min, *p* < 0.01), and immobilization duration was significantly prolonged (6.8 ± 2.1 vs. 4.2 ± 1.5 days, *p* < 0.001) (Table 1).

The correlation analyses between the surgery duration and immobilization duration of patients with DVT/PE are shown in Table 2. Surgery duration showed a significant positive correlation with diabetes mellitus (r = 0.459, *p* = 0.001), chronic kidney disease (r = 0.774, *p* = 0.021), platelet count (r = 0.491, *p* = 0.009), neutrophil count (r = 0.654, *p* = 0.005), glucose (r = 0.682, *p* = 0.004), CRP (r = 0.498, *p* = 0.010), and D-dimer levels (r = 0.659, *p* = 0.003). Conversely, negative correlations were observed between surgery duration and prophylactic anticoagulation (r = –0.412, *p* = 0.019), lymphocyte count (r = –0.586, *p* = 0.007), and serum calcium levels (r = –0.498, *p* = 0.012). It demonstrated strong positive correlations with glucose (r = 0.749, *p* = 0.001), neutrophil count (r = 0.731, *p* = 0.002), and D-dimer levels (r = 0.744, *p* = 0.002). On the other hand, age (r = –0.501, *p* = 0.026), hypertension (r = –0.542, *p* = 0.046), and chronic kidney disease (r = –0.846, *p* = 0.013) were inversely correlated with immobilization duration (Table 2).

In the parsimonious multivariate logistic regression model, five independent predictors of postoperative thromboembolic events were identified (Table 3). Advanced age was significantly associated with an increased risk of DVT/PE (OR = 1.44, 95% CI: 1.27–1.78, *p* = 0.003). The presence of diabetes mellitus (OR = 2.88, 95% CI: 2.71–3.00, *p* = 0.046) and chronic kidney disease (OR = 2.33, 95% CI: 2.11–2.67, *p* = 0.014) were also strong predictors. Among laboratory markers, elevated D-dimer levels were independently associated with nearly a two-fold increased risk (OR = 2.15, 95% CI: 1.88–2.52, *p* = 0.019). Procedural factors also played an important role: immobilization duration longer than 3 days conferred a more than two-fold higher risk of thromboembolic complications (OR = 2.21, 95% CI: 1.85–2.64, *p* = 0.028) (Table 3).

**Figure 2:**
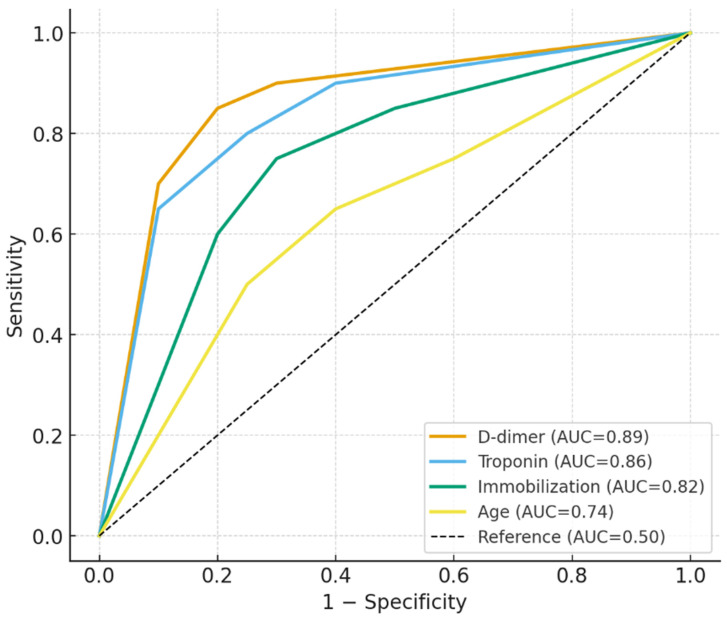
ROC curves for the most relevant predictors of postoperative DVT/PE (D-dimer, Troponin, Immobilization duration, and Age).

ROC analysis results for patients with DVT/PE are shown in Table 4. Neutrophil count >15.5 × 10^3^/μL yielded an AUC of 0.81 (95% CI: 0.74–0.88), with 78% sensitivity and 74% specificity (*p* = 0.009). D-dimer levels above 0.9 mg/L showed the strongest discriminative power, with an AUC of 0.89 (95% CI: 0.82–0.94), 88% sensitivity, and 84% specificity (*p* = 0.003). Troponin > 0.5 U/L also performed well, with an AUC of 0.86 (95% CI: 0.78–0.93), 85% sensitivity, and 80% specificity (*p* = 0.005). Immobilization duration exceeding 3 days had high predictive value, with an AUC of 0.82 (95% CI: 0.76–0.88), 80% sensitivity, and 75% specificity (*p* = 0.012). Similarly, glucose levels >180 mg/dL (AUC = 0.79, *p* = 0.017) and CRP > 7 mg/L (AUC = 0.76, *p* = 0.025) were significantly associated with DVT/PE risk. Age > 65 years, surgery duration > 120 min, and calcium levels < 8.8 mg/dL also showed moderate diagnostic performance with statistically significant AUC values (ranging from 0.68 to 0.74, *p* < 0.05). Platelet count < 350 × 10^3^/μL did not show statistically significant predictive value (*p* = 0.080) (Table 4, Figure 2).

## 4. Discussion

This study provided important insights into the incidence and risk factors associated with DVT and PE in patients undergoing orthopedic surgery. Among 300 patients, we observed a 16.7% incidence of postoperative DVT/PE, with advanced age, obesity, diabetes mellitus, and chronic kidney disease emerging as prominent risk factors. Additionally, prolonged immobilization, elevated inflammatory markers, increased glucose, and higher D-dimer levels were significantly associated with thromboembolic complications. In the refined multivariate logistic regression model, five predictors—advanced age, diabetes mellitus, chronic kidney disease, elevated D-dimer, and prolonged immobilization—were identified as the most reliable independent risk factors. These findings emphasize the multifactorial pathogenesis of thromboembolic events in orthopedic patients and underscore the critical need for comprehensive risk stratification and individualized prophylactic strategies in perioperative management.

In our ROC analysis, the cutoff value identified for neutrophil count (>15.5 × 10^3^/μL) may appear relatively high compared to thresholds commonly encountered in routine clinical practice. This value was determined using Youden’s index to maximize both sensitivity and specificity within our study population, rather than reflecting an established clinical benchmark. Previous studies have reported varying neutrophil thresholds associated with thromboembolic complications, highlighting that these values are highly dependent on patient demographics, comorbidities, and methodological approaches. Therefore, while an elevated neutrophil count clearly indicates systemic inflammation and a prothrombotic state, the specific cutoff obtained in our study should be interpreted cautiously and considered exploratory. Larger multicenter studies are warranted to validate clinically applicable neutrophil thresholds for thromboembolic risk prediction in orthopedic patients.

We found that advanced age, diabetes mellitus, and chronic kidney disease were independently associated with a higher risk of DVT/PE. These associations are in agreement with previous studies by Akrivou et al., who emphasized that aging and chronic diseases disrupt endothelial integrity and promote hypercoagulability, particularly in the surgical setting [11]. Similarly, Pastori et al. discussed the synergistic effect of comorbidities, such as diabetes and renal dysfunction, in enhancing VTE susceptibility via inflammatory and metabolic dysregulation [13].

Our data also showed that patients who did not receive pharmacologic prophylaxis had a two-fold increased risk of DVT/PE, supporting findings from Singjie et al., who reported that thromboprophylaxis with anticoagulants significantly reduces postoperative thromboembolic events in orthopedic patients [1]. In line with this, Zambelli et al. and Ghasemi et al. advocated for the routine use of LMWH or DOACs in high-risk individuals [3,17].

The elevation of inflammatory markers (neutrophils, CRP, and procalcitonin, which was measured selectively in postoperative patients with clinical suspicion) and metabolic parameters (glucose, D-dimer) in our study supports their predictive utility for thrombotic complications. However, the role of procalcitonin should be interpreted with caution since it was not uniformly available for all patients. Bilgin et al. previously highlighted the prognostic value of such markers in patients with PE, noting that neutrophilia and hyperglycemia reflect systemic inflammation and endothelial activation [5]. Our regression and ROC analyses confirmed the high diagnostic performance of these markers, with D-dimer > 0.9 mg/L and neutrophils > 15.5 × 10^3^/μL yielding AUCs of 0.89 and 0.81, respectively.

Lymphopenia was another independent risk factor observed in our study. This finding supports observations by Hu et al., who reported that lower lymphocyte counts are associated with a heightened risk of VTE, likely due to impaired immunoregulation in the hyperinflammatory state of surgery [5]. Furthermore, lower serum calcium and albumin levels in patients with thromboembolism suggest nutritional and systemic stress-related contributions to coagulation activation, as discussed by Dahl et al. [12].

Notably, troponin emerged as a strong predictor of DVT/PE in both regression and ROC models, with levels > 0.5 U/L providing an AUC of 0.86. While typically associated with myocardial injury, recent research by Bilgin et al. and van der Hulle et al. has linked elevated troponin to right ventricular strain in PE and systemic vascular stress [6,21].

Our analysis also underscores the role of procedure-related variables. Prolonged surgery and immobilization durations were significant predictors of DVT/PE, consistent with observations from Cao et al., who noted that venous stasis due to operative duration and delayed mobilization substantially increases thrombosis risk [22]. ROC analysis revealed that immobilization exceeding 3 days had high diagnostic accuracy (AUC: 0.82), emphasizing the critical role of early mobilization.

Our parsimonious multivariate model, which included only variables supported by both univariate analysis and clinical relevance, demonstrated that age, diabetes mellitus, chronic kidney disease, elevated D-dimer, and prolonged immobilization were the most reliable independent predictors of postoperative DVT/PE. These results are in line with previous reports indicating that advanced age and chronic comorbidities disrupt endothelial function and enhance prothrombotic states. Elevated D-dimer reflects increased fibrin turnover and has been widely validated as a sensitive biomarker for venous thromboembolism risk. Similarly, prolonged immobilization remains a well-established procedural risk factor due to venous stasis. By focusing on these key predictors, our analysis avoids model overfitting and provides a clinically applicable framework for perioperative risk stratification. Importantly, these results underscore the need for more intensive thromboprophylaxis and early mobilization strategies in patients presenting with this high-risk profile. Furthermore, our findings align with recent analyses by Tsai et al. and Kollapaneni et al., who proposed incorporating both metabolic and procedural risk profiles into thrombosis prediction models [19,20].

Compared to previously published studies, the present study offers a more comprehensive evaluation by integrating demographic, clinical, laboratory, and procedural parameters in a single predictive model for DVT/PE after orthopedic surgery. While previous studies primarily focused on the effectiveness of pharmacologic prophylaxis, our study additionally highlights the predictive value of inflammatory and metabolic biomarkers such as neutrophil count, D-dimer, glucose, and CRP levels. Furthermore, unlike prior reports that examined VTE incidence in specific procedures (e.g., total knee arthroplasty or hip fracture), our cohort included a wider spectrum of orthopedic interventions, enhancing the generalizability of the findings. The inclusion of troponin as a novel predictor and the use of ROC analysis to determine optimal diagnostic thresholds also distinguish our study from previous investigations, offering practical tools for clinical risk stratification.

### Limitations of the Study

This study has some limitations that should be acknowledged. First, the retrospective design inherently carries a risk of selection bias and limits the ability to establish causality. Data collection relied on existing hospital records, which may have included incomplete or inconsistent entries. Second, the diagnosis of DVT and PE was based on imaging methods (venous Doppler ultrasonography and thoracic CT angiography) performed only in patients with clinical suspicion. Asymptomatic cases that did not undergo imaging may therefore have been missed, potentially leading to underestimation of the true incidence and a risk of misclassification when dividing patients into DVT/PE (+) and (−) groups. Third, most demographic, clinical, and laboratory parameters were obtained from preoperative records; however, due to the retrospective design, we could not confirm complete uniformity regarding the timing of all measurements, nor could we assess whether modifiable factors (such as glycemia or renal parameters) were corrected before surgery. Fourth, although multiple laboratory and clinical parameters were evaluated, the relatively limited number of thromboembolic events restricted the number of variables that could be included in the multivariate logistic regression analysis. To address this, we applied a stepwise variable selection approach to minimize the risk of model overfitting and to ensure the robustness of the results. Fifth, although multiple laboratory and clinical parameters were evaluated, the relatively limited number of thromboembolic events restricted the number of variables that could be included in the multivariate logistic regression analysis. Furthermore, procalcitonin was not a routine preoperative test and was measured only postoperatively in patients with clinical suspicion of infection or inflammation. Therefore, its availability was limited, and its predictive value in this cohort should be interpreted with caution. Sixth, the reasons why some patients did not receive pharmacologic thromboprophylaxis (e.g., bleeding risk, comorbidities, physician or institutional preferences) and the determinants of immobilization duration (e.g., surgical complexity, postoperative complications, patient-related factors, or organizational issues) could not be systematically analyzed due to the retrospective design. Finally, the study did not account for potential confounders such as preoperative mobility status, genetic thrombophilia, or intraoperative blood loss, which could influence thromboembolic risk. Prospective, multicenter studies with standardized diagnostic protocols and larger sample sizes are warranted to validate these findings and enhance their applicability in clinical practice.

## 5. Conclusions

In conclusion, this study demonstrated that deep vein thrombosis and pulmonary embolism remain significant postoperative complications following orthopedic surgery, with a multifactorial etiology involving patient-related, inflammatory, metabolic, and procedural factors. Advanced age, diabetes mellitus, chronic kidney disease, lack of prophylactic anticoagulation, and elevated levels of neutrophils, D-dimer, glucose, and CRP were identified as independent predictors of thromboembolic events. Additionally, prolonged immobilization emerged as a modifiable and clinically important factor. These findings highlight the importance of individualized risk assessment and timely implementation of pharmacologic and mechanical prophylaxis. The incorporation of laboratory biomarkers into perioperative evaluation may improve early identification of high-risk patients. Prospective studies are required to validate these findings and to develop standardized risk prediction tools for clinical use.

## Figures and Tables

**Table 1:** Comparison of sociodemographic characteristics and laboratory findings of patients.

Variable	DVT/PE (−) (n = 250)	DVT/PE (+) (n = 50)	*p*-Value
	Mean ± SD or n (%)		
Age (years)	65.2 ± 10.1	72.5 ± 8.7	<0.001
Gender (Male/Female)	138 (55.2%)/112 (44.8%)	33 (66%)/17 (34%)	0.02
BMI (kg/m^2^)	28.3 ± 4.5	32.1 ± 5.3	<0.001
Diabetes Mellitus	50 (20%)	20 (40%)	<0.01
Hypertension	88 (35.2%)	25 (50%)	<0.01
Chronic Kidney Disease	25 (10%)	12 (24%)	<0.001
Prophylactic Anticoagulation	213 (85.2%)	23 (46%)	<0.001
WBC (×10^3^/µL)	7.5 ± 1.8	9.2 ± 2.0	<0.01
RDW (%)	13.1 ± 1.2	15.4 ± 1.5	<0.001
Platelet (×10^3^/µL)	250 ± 60	280 ± 75	<0.05
Neutrophil (×10^3^/µL)	4.5 ± 1.2	6.1 ± 1.5	<0.01
MPV (fL)	9.8 ± 1.5	11.2 ± 1.8	<0.05
Monocyte (×10^3^/µL)	0.6 ± 0.2	0.9 ± 0.3	<0.001
Lymphocyte (×10^3^/µL)	1.8 ± 0.6	1.2 ± 0.4	<0.01
Ca++ (mg/dL)	9.1 ± 0.5	8.5 ± 0.6	0.04
Glucose (mg/dL)	105 ± 18	125 ± 22	<0.001
Albumin (g/dL)	4.1 ± 0.6	3.5 ± 0.7	0.02
BUN (mmol/L)	14.2 ± 5.5	18.5 ± 6.2	<0.01
Creatinine (mg/dL)	0.9 ± 0.3	1.3 ± 0.5	0.05
Troponin (U/L)	0.03 ± 0.01	0.12 ± 0.05	<0.01
D-dimer (mg/L)	0.5 ± 0.3	1.8 ± 0.6	<0.001
CRP (mg/L)	3.2 ± 1.1	6.5 ± 2.3	<0.001
Procalcitonin (ng/L)	0.04 ± 0.02	0.10 ± 0.04	<0.01
DVT Incidence	0 (0%)	50 (100%)	-
PE Incidence	0 (0%)	25 (50%)	-
Surgery Type (TKA/THA/Others)	113 (45.2%)/88 (35.2%)/49 (19.6%)	25 (50.0%)/15 (30.0%)/10 (20.0%)	0.04
Surgery Duration (min)	110 ± 25	135 ± 30	<0.01
Immobilization Duration (days)	4.2 ± 1.5	6.8 ± 2.1	<0.001

DVT: Deep Vein Thrombosis, PE: Pulmonary Embolism, BMI: Body Mass Index, TKA: Total Knee Arthroplasty, THA: Total Hip Arthroplasty, WBC: White Blood Cell, RDW: Red Cell Distribution Width, MPV: Mean Platelet Volume, BUN: Blood Urea Nitrogen, CRP: C-Reactive Protein.

**Table 2:** The correlation analyses between the surgery duration and immobilization duration of patients with DVT/PE.

Parameters	Surgery Duration r Value, *p* Value	Immobilization Duration r Value, *p* Value
Age (years)	−0.378, 0.041	−0.501, 0.026
BMI (kg/m^2^)	−0.350, 0.043	−0.179, 0.004
Diabetes Mellitus	0.459, 0.001	0.511, 0.015
Hypertension	0.275, 0.026	−0.542, 0.046
Chronic Kidney Disease	0.774, 0.021	−0.846, 0.013
Prophylactic Anticoagulation	−0.412, 0.019	−0.528, 0.014
WBC (×10^3^/µL)	0.312, 0.017	0.429, 0.022
RDW (%)	−0.215, 0.035	−0.367, 0.029
Platelet (×10^3^/µL)	0.491, 0.009	0.572, 0.006
Neutrophil (×10^3^/µL)	0.654, 0.005	0.731, 0.002
MPV (fL)	−0.362, 0.028	−0.415, 0.014
Monocyte (×10^3^/µL)	0.245, 0.033	0.369, 0.019
Lymphocyte (×10^3^/µL)	−0.586, 0.007	−0.642, 0.003
Ca++ (mg/dL)	−0.498, 0.012	−0.519, 0.011
Glucose (mg/dL)	0.682, 0.004	0.749, 0.001
Albumin (g/dL)	−0.476, 0.009	−0.531, 0.008
BUN (mmol/L)	0.357, 0.024	0.441, 0.018
Creatinine (mg/dL)	0.221, 0.031	0.332, 0.027
Troponin (U/L)	0.722, 0.002	0.813, 0.001
D-dimer (mg/L)	0.659, 0.003	0.744, 0.002
CRP (mg/L)	0.498, 0.010	0.589, 0.007
Procalcitonin (ng/L)	0.331, 0.021	0.425, 0.017

**Table 3:** Parsimonious multivariable logistic regression for predictors of postoperative DVT/PE (n = 300; events = 50).

Variable	DVT/PE		
	Odds Ratio	95% CI	*p* Value
Age (years)	1.44	1.27–1.78	0.003
Diabetes Mellitus	2.88	2.71–3.00	0.046
Chronic Kidney Disease	2.33	2.11–2.67	0.014
D-dimer (mg/L)	2.15	1.88–2.52	0.019
Immobilization Duration (days)	2.21	1.85–2.64	0.028

**Table 4:** ROC analysis results in patients with DVT/PE.

Parameters	Cut-Off	Sensitivity	Specifity	AUC (95% CI)	*p* Value
Age (years)	65<	0.72	0.68	0.74 (0.66–0.82)	0.021
Platelet (×10^3^/µL)	350<	0.60	0.55	0.65 (0.57–0.72)	0.080
Neutrophil (×10^3^/µL)	15.5	0.78	0.74	0.81 (0.74–0.88)	0.009
Lymphocyte (×10^3^/µL)	5.5	0.69	0.70	0.72 (0.65–0.79)	0.032
Ca++ (mg/dL)	8.8<	0.64	0.66	0.68 (0.60–0.76)	0.045
Glucose (mg/dL)	180<	0.76	0.72	0.79 (0.72–0.85)	0.017
Troponin (U/L)	0.5<	0.85	0.80	0.86 (0.78–0.93)	0.005
D-dimer (mg/L)	0.9<	0.88	0.84	0.89 (0.82–0.94)	0.003
CRP (mg/L)	7<	0.74	0.71	0.76 (0.69–0.82)	0.025
Surgery Duration (Min.)	120<	0.70	0.68	0.72 (0.65–0.78)	0.038
Immobilization Duration	3<	0.80	0.75	0.82 (0.76–0.88)	0.012

## Data Availability

The original contributions presented in the study are included in the article, further inquiries can be directed to the corresponding author.
